# Fistulating Intraductal Papillary Mucinous Neoplasms (IPMNs): Case Series and Discussion of a Rare Complication

**DOI:** 10.3390/jcm15093255

**Published:** 2026-04-24

**Authors:** Guanqi Hang, Logaswari M, Shuyi Guo, Emma Choon Hwee Lee, Yang Shan Edmond Lim, Zhuyi Rebekah Lee

**Affiliations:** 1 Department of Cardiothoracic and Abdominal Radiology, Singapore General Hospital, Singapore 169608, Singapore; 2 Department of Pathology, Singapore General Hospital, Singapore 169608, Singapore

**Keywords:** pancreas, intraductal papillary mucinous neoplasm, fistula

## Abstract

**Background**: Intraductal papillary mucinous neoplasm (IPMN) is a mucin-producing pancreatic tumor with variable malignant potential. While most are asymptomatic and indolent, a subset progress to invasive carcinoma or cause local complications such as pancreatitis. Spontaneous fistulation into adjacent organs is an increasingly recognized phenomenon with impact on prognosis and management. The incidence of fistulation in IPMN in the reported literature is 1.9–6.6%. The most common sites are the stomach, duodenum and bile duct. Reported outcomes are poor, with a median survival of approximately 16 months. **Methods**: We describe four patients with IPMN complicated by fistula, confirmed by endoscopic or histopathological evaluation with CT and MRI images and discuss the available literature of fistulating IPMN. **Results**: Fistulation occurred at the common bile duct, stomach, duodenum and duodeno-jejunal junction. Two of four patients passed away at 4.8 and 24.8 months from detection of fistula. Histology revealed high-grade dysplasia or invasive carcinoma in most patients, highlighting the aggressive nature of IPMNs complicated by fistulae. **Conclusions**: Our findings reinforce the importance of recognizing fistula formation as a marker of aggressive disease in IPMN. Although surgical resection remains the treatment of choice in suitable candidates, the rarity of this entity means that standardized management guidelines are lacking.

## 1. Introduction 

Intraductal papillary mucinous neoplasm (IPMN) is a cystic lesion characterized by a proliferation of mucin-producing epithelial cells with resultant dilatation of the pancreatic ducts [1,2]. Overall detection of pancreatic cysts is 15% on MRI compared to 3% on CT, with increasing incidence in the older age group [3]. Of these, IPMNs account for almost 50% of incidentally detected cystic lesions [4].

Most IPMNs demonstrate benignity or indolent course and are managed conservatively. However, the clinical significance of IPMN lies in its malignant potential, which may develop into invasive carcinoma that necessitates surgical management [5,6]. As such, radiological surveillance plays a central role in risk assessment and patient selection for surgery. The most commonly cited international consensus guidelines, the Fukuoka guidelines, were first proposed by Tanaka et al. in 2006 [5] with the latest revision in 2024. Much ongoing research is directed at refining risk stratification and surveillance strategies [1,2].

IPMNs are classified into main duct (MD-IPMN), branch duct (BD-IPMN), and mixed types according to their involvement of the pancreatic ductal system [7,8]. Malignant potential differs across subtypes, with BD-IPMNs demonstrating a risk of malignancy of 19–30%, while MD-IPMNs exhibit substantially higher rates, reported at 40–70% [1,2,5,6,8,9,10]. IPMNs are also classified into low-grade, moderate, or high-grade dysplasia, and invasive carcinoma based on the pathology [11]. 

Although IPMNs generally carry a relatively favorable prognosis, cases complicated by fistula formation have been reported to confer a poor outcome, resembling the prognosis of pancreatic intraductal adenocarcinoma [12,13]. Fistula formation into adjacent organs represents an uncommon but distinctive complication of IPMN, with a reported prevalence ranging from 1.9% to 6.6% among affected patients [12,14]. Given the rarity of this complication, we present a case series of patients with IPMN complicated by fistula formation involving various organs. The requirement for institutional review board approval was waived, and verbal informed consent was obtained from all patients. 

## 2. Materials and Methods

### 2.1. Patient Selection

This retrospective case series was conducted at a single tertiary institution from August 2023 to July 2025. Informed consent to use deidentified data was obtained from all patients.

Four patients with histologically confirmed IPMN were included for review. Cases were voluntarily submitted during an internal call within the department for cases that fit the inclusion criteria. The inclusion criteria were:

1. Confirmed diagnosis of IPMN on histopathological examination.
2. Definitive fistulation on diagnostic CT or MRI imaging at our institution.
3. Minimum 24 months follow-up.

Cases were not consecutively enrolled and were selected for illustrative and educational purposes rather than epidemiologic representation.

### 2.2. Imaging Protocol

CT examinations: Post-contrast-enhanced CT examinations were performed on multidetector scanners (Siemens SOMATOM Force, Siemens Healthineers, Erlangen, Germany) with automated tube current modulation. Images were reconstructed in 3 mm thickness in axial and coronal phases, in the portovenous phase.

MRI examinations: Performed on 1.5T or 3T systems (Siemens MAGNETOM Avanto Fit/MAGNETOM Vida/Skyra, Siemens Healthineers, Erlangen, Germany) with standard hepatobiliary/pancreatic protocols, including:

- Axial and coronal T2-weighted imaging;
- In- and out-phase T1-weighted imaging;
- Diffusion weighted imaging (DWI) with b-values up to 1000;
- Dynamic contrast enhancement on fat saturated T1-weighted imaging;
- Steady-state free precession (SSFP) imaging of the biliary tree and pancreatic ducts.

### 2.3. Analysis

The index scan is considered the first CT or MRI demonstrating a fistula. The following tumor characteristics were assessed:

- Tumor size and location;
- Enhancement characteristics;
- Involvement of the main pancreatic duct (MPD);
- Involvement of adjacent structures.

Clinical data including patient demographics, presenting symptoms, laboratory markers, histopathological findings, surgical and non-surgical interventions, and mortality (if applicable) of patients were obtained from our institution’s electronic medical records. Given the small sample size and illustrative intent, statistical analysis was not performed.

## 3. Results

Four cases were identified for evaluation, with patients in the 6–7th decade of life. There was no obvious predilection for location within the pancreas and the average tumor volume was 77 cc. All cases involved the main pancreatic duct (MPD) and were deemed main duct or mixed-type IPMN, with three cases demonstrating solid enhancing component on post-contrast CT or MRI.

The fistulas involved the common bile duct (*n* = 1), stomach (*n* = 1), proximal duodenum (*n* = 1) and duodeno-jejunal junction (*n* = 1). Three cases were surgically resected, while one patient was not a surgical candidate due to comorbidities. Of the three cases with complete histopathological evaluation after resection, all showed background chronic pancreatitis; only two showed invasive carcinoma at the fistula edge. Two of three resected lesions showed underlying chronic pancreatitis. Three cases demonstrated biliary obstruction and raised serum bilirubin; the IPMN caused a direct mass effect on the common bile duct (CBD) for case 3, while biliary obstruction was due to excessive mucin production and MPD dilatation in cases 1 and 2. One patient succumbed to pancreatic disease 4.8 months from the index study, while another passed away after a non-eventful post-operative period from cardiovascular disease at 24.8 months (Table 1).

### 3.1. Case 1: Fistula to Common Bile Duct (CBD)

**Figure 1:**
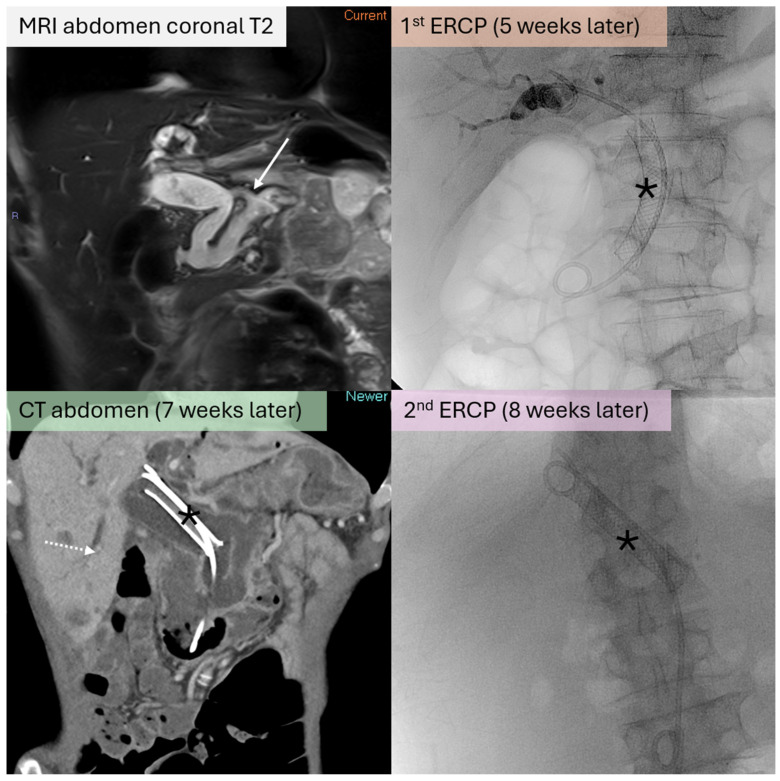
(**Top left**) Surveillance MRCP study (coronal T2 sequence) showing fistulous communication between dilated MPD (white arrow) and upper CBD. (**Top right**) Plastic double pigtail and fully covered metallic (black asterisk) biliary stents placed endoscopically during endoscopic retrograde cholangiopancreatography (ERCP). (**Bottom left**) Repeat CT performed for obstructive jaundice showed proximal migration of the covered metallic stent (black asterisk) with resultant intrahepatic biliary dilatation (dashed white arrow). (**Bottom right**) Repeat ERCP confirmed proximal migration of the metallic stent (black asterisk). Stent was removed and exchanged (not pictured).

A 79-year-old Chinese male patient, with a known history of mixed-type IPMN under routine surveillance, presented with progressive jaundice and abdominal discomfort. Imaging revealed an ill-defined soft tissue mass in the pancreatic body associated with marked dilatation of both the MPD and the biliary system. Further evaluation with MR cholangiopancreatography (MRCP) demonstrated the formation of a fistula between the MPD and the CBD (Figure 1). The patient was deemed too weak for surgical resection and was palliated with endoscopic CBD stenting. However, excessive mucin production caused CBD stent migration and mechanical obstruction of the previously placed stents, requiring repeat endoscopic stent exchange. Despite this, the disease progressed with the development of hepatic metastases, and the patient eventually succumbed to the illness within 5 months of fistula formation.

### 3.2. Case 2: Fistula to the Stomach

**Figure 2:**
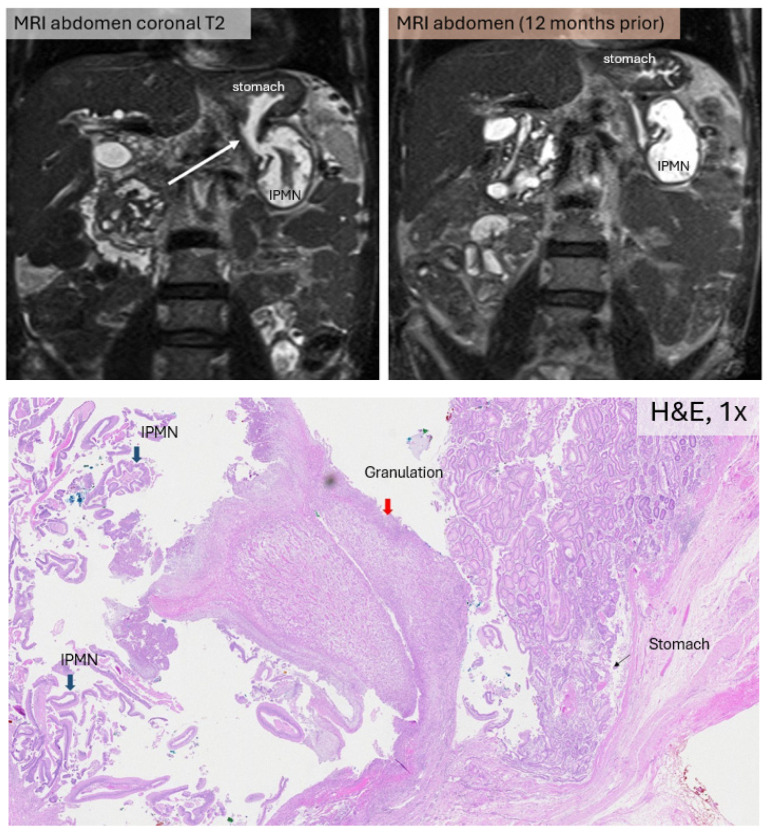
(**Top left**) Surveillance MRI abdomen (coronal T2 sequence) showing fistulous communication between markedly dilated MPD and inferior gastric fundus (white arrow). (**Top right**) Earlier MRI abdomen showing an intact gastric wall near the dilated MPD. (**Bottom**) Histopathological image of IPMN (thick blue arrow) eroding into stomach (thin black arrow) with intervening sliver of granulation tissue (red arrow), H&E, 1×.

A 69-year-old Chinese male patient with a known history of multiple small main duct IPMNs presented with weight loss. MRCP was done and found to have a markedly dilated pancreatic duct containing solid enhancing components, raising suspicion for malignancy. Imaging further demonstrated a fistulous tract between the MPD and the gastric fundus at the distal pancreatic body (Figure 2). While there was no involvement of the colon, there was extensive mesenteric involvement compromising the left colic vessels. Given the extent of the disease, the patient underwent a total pancreatoduodenectomy with splenectomy, left hemicolectomy and near-total gastrectomy. Histopathological examination confirmed pancreatic IPMN with areas of high-grade dysplasia. His immediate post-operative period was uneventful, but his recovery was marked by recurring episodes of labile blood glucose and he eventually passed away 25 months later from cardiovascular disease.

### 3.3. Case 3: Fistula to the Proximal Duodenum

**Figure 3:**
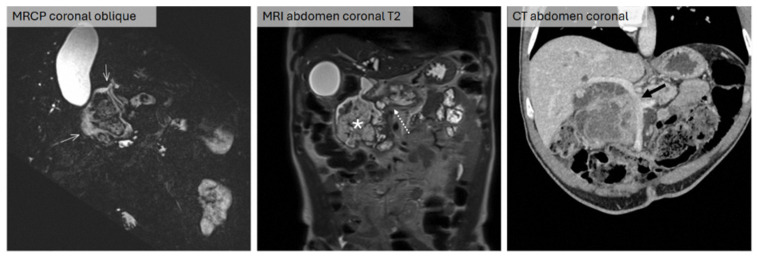
(**Left**) Surveillance MRI abdomen (coronal oblique, 0.6 mm SSFP sequence) shows multiple fistulas between the mass and the proximal duodenum (white arrows). (**Middle**) Same MRI abdomen (coronal T2 sequence) showing dilated MPD (white dashed arrow) with multiseptated T2 hyperintense mass lesion in the pancreatic head compatible with IPMN (white asterisk). (**Right**) Contrast-enhanced CT abdomen (coronal) showing involvement of the adjacent SMV and portal vein (black arrow).

A 61-year-old male Chinese patient with a history of gallstones presented with right hypochondrial pain. CT abdomen and pelvis performed for cholecystitis showed a large solid-cystic lesion arising from the pancreatic head and uncinate process, associated with biliary dilatation and a mass involving the superior mesenteric vein (SMV) and portal vein confluence. Further evaluation with MRCP revealed the presence of direct erosion of the lesion into the duodenum with multiple fistulous tracts (Figure 3). An extended total pancreatosplenectomy with en bloc right hemicolectomy and portal vein/SMV resection were performed. Histopathological analysis confirmed a colloid carcinoma arising from an IPMN with direct extension into the duodenum; marked acute on chronic pancreatitis and abscess formation was seen in the background pancreatic parenchyma. Post-operatively, the patient recovered well and is currently under routine follow-up.

### 3.4. Case 4: Fistula to Duodeno-Jejunal Junction

**Figure 4:**
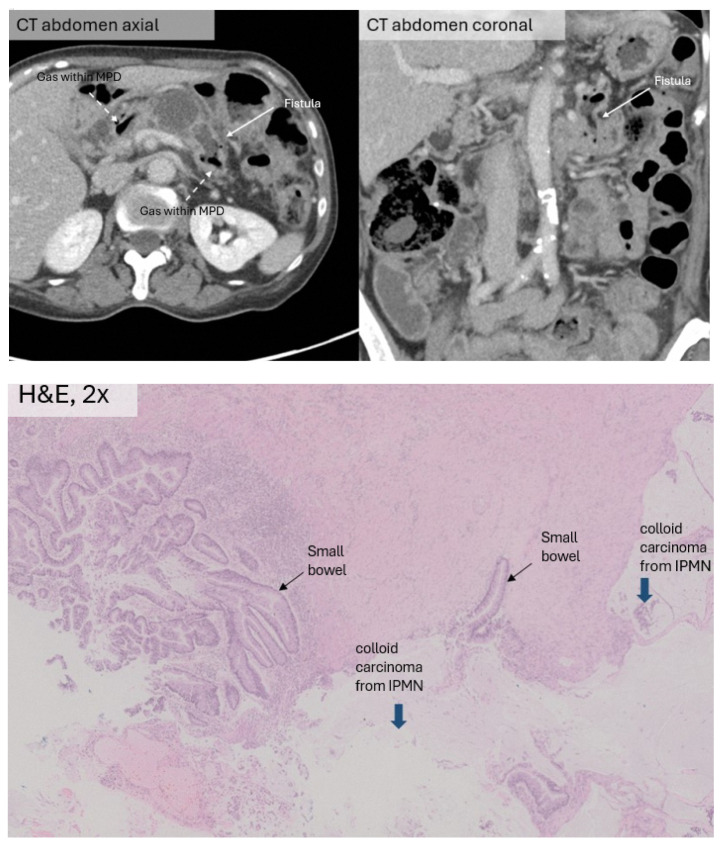
(**Top left**) Contrast-enhanced CT (axial) demonstrating air in the MPD (dashed arrows) with fistulous connection to the duodeno-jejunal junction (solid white arrow). (**Top right**) Same contrast-enhanced CT (coronal) showing communication with the gas containing jejunal loop. (**Bottom**) Histopathological image showing mucin-rich colloid carcinoma (thick blue arrows) arising from the IPMN invading into small bowel (thin black arrows), H&E, 2×.

A 73-year-old Chinese woman with a history of hemicolectomy for colon cancer was incidentally found to have a dilated MPD on surveillance CT. ERCP and endoscopic biopsy demonstrated a main duct IPMN, and histology confirmed high-grade dysplasia. The patient was offered up-front surgical resection but declined at the time. On routine follow-up, serial CT imaging revealed progressive dilatation of the MPD and new development of a fistulous connection between the duct and the duodeno-jejunal junction. After repeat discussion with the patient, she underwent neoadjuvant chemotherapy and eventual total pancreatectomy and splenectomy. Histology showed IPMN with an associated invasive colloid carcinoma (Figure 4). 

## 4. Discussion

### 4.1. Incidence

Fistula formation is a rare complication of IPMN but has been increasingly reported in recent years, likely due to advances in cross-sectional imaging. In one review comprising 54 articles (36 case reports, three retrospective analyses, and 15 pictorial reviews), fistulation was identified in 83 patients involving a total of 119 organs [15]. Among the organs affected by IPMN-related fistulation, the stomach is most frequently involved (34%), followed by the duodenum (30%), bile duct (25%), and, less commonly, the colon (5%), small intestine, spleen, portal vein, or even chest wall [15]. Multiorgan involvement is reported in up to 39% [15]. Many fistulizing IPMNs are of the intestinal subtype (producing abundant mucin), and fistula formation often signals more aggressive biology, with a higher likelihood of high-grade dysplasia or invasive cancer. Fistulation into solid organs is rarer. It has been suggested that solid organs better resist the effects of elevated pressure of mucin production, one of the mechanisms considered to contribute to fistula development [16].

### 4.2. Pathophysiology

The pathogenesis of fistula formation in IPMN remains incompletely understood. Several mechanisms have been proposed in the literature, including:

(1) direct tumor invasion, associated with invasive carcinoma (e.g., mucinous adenocarcinoma) or high-grade dysplasia, where the neoplasm infiltrates surrounding tissue;
(2) mechanical penetration driven by elevated intraductal pressure from a combination of abundant mucin production, ductal obstruction, or cystic expansion causing erosion into adjacent structures without overt malignant invasion around the fistula site;
(3) chronic inflammation and autodigestion by pancreatic enzymes causes recurrent pancreatitis that weakens tissue planes. Inflammatory remodeling promotes adhesion and fistula tract formation [12,17].

Yamada et al. [17] further proposed that mechanical and enzymatic mechanisms often act synergistically: increased intraductal pressure first erodes the ductal epithelium, and enzymatic autodigestion promotes dissolution of the duct wall and parenchyma. Subsequent erosion into peripancreatic fat leads to inflammation and adhesion to adjacent organs, worsening autodigestive injury to these organs, culminating in fistula formation. This sequence illustrates how chronic mucin overproduction, inflammation, and enzymatic activity can progressively weaken anatomical barriers, allowing pathological communication with surrounding luminal structures.

As such, the presentation of fistulizing IPMN includes symptoms of recurrent acute pancreatitis, abdominal pain, jaundice (if the biliary tree if affected), abscess and gastrointestinal bleeding. Pain and pancreatitis may paradoxically improve after fistulization due to the natural decompression of mucin into adjacent luminal structures.

### 4.3. Diagnosis and Assessment

When fistulating IPMN is suspected, diagnostic CT is typically the first line for evaluation due to ready availability. Where available, MRI and MRCP demonstrate superior soft tissue resolution and can more clearly delineate the abscess and fistulous tracts. There are a wider variety of benign and malignant cystic lesions that may fistulate to other organs, mimicking fistulizing IPMN (Table 2).

Ultimately, endoscopy with ultrasound (EUS) may be required to obtain histological sample. Endoscopy also may be therapeutic in treating fistulation to the upper gastrointestinal organs (clip closure) or relief of obstruction (e.g., placing biliary drains).

Despite the limited sample size, histological findings for our case series corroborate this theory of mixed pathogenesis, with two of three of the surgically resected tumors demonstrating chronic pancreatitis. Similarly, two out of three cases showed direct tumor invasion. This highlights the heterogeneity of tumor biology and supports the theory of fistula development as a multifactorial phenomenon. 

### 4.4. Management and Surveillance

Because IPMN is generally considered a slow-growing tumor with a favorable prognosis, radiological risk stratification remains essential to avoid unnecessary morbid surgery. According to the international Fukuoka consensus guidelines, high-risk stigmata for malignancy in IPMN include a MPD diameter of ≥10 mm, the presence of an enhancing mural nodule > 5 mm, and evidence of biliary obstruction [5,19].

Guidelines suggest that patients with high-risk stigmata that are surgically fit candidates should undergo resection. Those with worrisome features such as cysts ≥ 3 cm, enhancing mural nodules < 5 mm, or a MPD diameter of 5–9 mm should be further evaluated with endoscopic ultrasound (EUS) and cytology. Surgical resection is recommended if EUS demonstrates mural nodules ≥ 5 mm, main duct involvement, or cytology suspicious or positive for malignancy. In the absence of these findings, patients should be monitored with cross-sectional imaging or EUS at intervals determined by cyst size [5,19]. 

Fistula formation is not currently included in these guidelines though most cases of fistulization occur in high-risk main duct IPMN [14]. Furthermore, the development of fistulae in IPMN has been associated with a significantly poorer prognosis. A retrospective study on 274 patients with IPMNs by Kobayashi et al. [12] reported a median survival of only 16 months in patients with fistula formation. By contrast, the 5-year survival of patients with non-invasive IPMN is estimated to be 90% [20]. This suggests that the presence of a fistula may be a marker of advanced disease poorer prognosis. 

Because of the rarity of reported fistulation in IPMN, a standardized treatment strategy has yet to be established. A review of 27 case reports of fistulating IPMN by AbuDalu shows 37% underwent surgical resection, and 15% rejected surgery or died prior to surgery; only 3% treated conservatively. However, surgical management is not entirely described in 44% of case studies [21]. Endoscopic intervention such as stenting, endoscopic closure of accessible fistulas or conservative management have been explored but are less common [21]. Management decisions are often individualized, considering the patient’s clinical status, the extent of fistulation and complications arising from fistulation, and the degree of dysplasia or invasive carcinoma identified [19,22].

While resection remains the mainstay for surgically fit patients, particularly those with high-risk or malignant features, the role of less invasive approaches and surveillance strategies remains uncertain. Further accumulation of case reports and larger cohort studies will be essential to establish evidence-based guidelines for this challenging clinical entity. 

### 4.5. Study Limitations

A significant limitation of our study is small sample size (n = 4) and the fact that it was selected for educational purposes, which poses inherent selection bias. This significantly limits the analysis of the epidemiology and prognosis of this disease. However, our study generally corroborates the findings in the wider literature, highlighting the diverse pattern of fistulation, the heterogeneity of the disease and the natural history of fistulating IPMN. Data gathered through a larger audit of fistulating IPMN established on histology and imaging is necessary to determine the true incidence and epidemiology of the disease.

## 5. Conclusions and Future Direction

Fistula formation is a rare but clinically significant complication of IPMN that is increasingly recognized with advances in cross-sectional imaging. Though small, this case series highlights the diverse anatomical sites of fistulation and demonstrates its relationship with high-grade dysplasia or invasive carcinoma.

The development of fistulae in IPMN may correlate with more aggressive disease and poorer prognosis. Given its rarity and the heterogeneity of histopathological findings, no standardized treatment strategy has been established. Larger studies are needed to clarify the epidemiology of disease, prognostic implications of fistulation and inform evidence-based guidelines for this challenging entity.

## Figures and Tables

**Table 1:** Summary of demographic data, tumor characteristics, laboratory markers, treatment, length of stay and demise (if applicable). MPD = main pancreatic duct.

#	Age/ Sex	Tumor Location	Tumor Size (cm)	Volume (cc)	MPD Involvement	Solid Enhancement	Structure Involved	Surgical Resection	Invasive Carcinoma at Fistula	Background Pancreatitis	High Bilirubin	Demise; Months from Index Scan
1	79/M	Body	6.7 × 3.1 × 3.9	42.7	Yes	Yes	Biliary tree	No	NA	NA	Yes	Yes; 4.8
2	69/M	Tail	5.9 × 2.7 × 6.7	55.8	Yes	Yes	Stomach (fundus)	Yes	No	Yes	Yes	Yes; 24.8
3	61/M	Head, uncinate	7.6 × 5.4 × 8.4	180	Yes	Yes	Proximal duodenum	Yes	Yes	Yes	Yes	No
4	73/F	Body	6.5 × 3.2 × 2.7	29.3	Yes	No	Duodeno-jejunal junction	Yes	Yes	No	No	No

**Table 2:** Comparison table of differential diagnoses for fistulating IPMN, which demonstrate similar imaging characteristics. Statistics taken from references [3,4,18].

Pathology	Imaging and Pathological Features	Patient Characteristics and Clinical Presentation	Similarities	Differences
**Intraductal papillary neoplasm (IPMN)**	Variable malignant potential. Branch duct 3–12%; main duct 33–85% malignant. Branch duct IPMN communicates with the MPD. Main duct IPMN cause direct dilatation of the MPD.	Equal gender distribution. 5th to 7th decade in life. May cause pancreatitis.	-	-
**Pancreatic ductal adenocarcinoma (PDAC)**	Malignant and aggressive, with 5-year survival of 10%. Invasive soft tissue mass causing upstream ductal obstruction. Typically, solid but relatively hypoenhancing relative to pancreatic parenchyma.	Slight male predominance. 50% metastatic at presentation.	Dilated upstream MPD, may present with similar clinical features (weight loss, abdominal pain).	Solid instead of cystic on CT, with higher attenuation. Less T2w hyperintense on MRI Aggressive with poor prognosis.
**Mucinous cystadenoma/cystadenocaricnoma neoplasm (MCN)**	Malignant potential 17.5%, with 5-year survival of 62%. Multilocular macrocystic pancreatic lesion, typically in the body and tail. Typically benign but may develop invasive adenocarinoma. Histology typically demonstrates ovarian-type stroma. Average size 10–12 cm.	Almost exclusively in women (90%). Occurs in 4th to 6th decade of life. Mostly asymptomatic.	High T2w signal, may be large with near cystic fluid density.	Single lesion with thick fibrous capsule, which may demonstrate calcifications.
**Serous cystadenoma**	Almost always benign (1% malignant potential). Microcystic (honeycomb) appearance; central scar (30% of cases); may be macrocystic or solid-appearing; no duct communication.	Predominantly in women (60–75%). 5th to 7th decade of life. Can present with jaundice, pancreatitis, abdominal pain, or palpable mass.	Macrocystic lesions may mimic IPMN.	Microcystic appearance (honeycomb) with central scar. No communication with main pancreatic duct.
**Intraductal tubulopapillary neoplasm (ITPN)**	Malignant potential 60–75%, but with favorable 5-year survival of 71%. Rare intraductal pancreatic lesion (3% of intraductal lesions). Isodense or mildly hypodense. Tumor cells form tubulopapillae which plug the MPD. No to little cytoplasmic mucin.	No clear sex predilection.	Dilated and irregular MPD.	Solid mass within the duct lumen, mucin poor. Higher attenuation compared to IPMN.
**Pancreatic intraepithelial neoplasm (PanIN)**	Premalignant with unknown malignant potential. Extremely common, found in 77–86% of autopsies. Precursor lesion to PDAC.	No clear sex predilection. Incidence increases with age and low-grade PanIN are ubiquitous in adults > 40 years.	Typical microcystic < 5 mm, flat, or not visible, but can cause MPD dilatation and parenchymal atrophy.	Usually smaller or entirely not visible.
**Pancreatic pseudocyst**	Benign. Post-pancreatitis cystic cavity containing autodigestive enzymes that may erode into stomach/duodenum.	Any age or gender. History of pancreatitis.	Large dilated cystic lesion, which can erode into adjacent organs.	History of recent pancreatitis or gallstones. May not communicate with MPD.
**Walled-off necrosis (WON)**	Benign. Chronic post-pancreatitis cystic cavity with thick walls containing autodigestive enzymes that may erode into stomach/duodenum.	Any age or gender. History of pancreatitis.	Can erode into stomach/duodenum.	Heterogeneous debris, pancreatic necrosis, pancreatitis history.
**Choledochal cyst or biliary dilation**	Benign. Congenital cystic dilatation of the biliary tree.	Rare, albeit more common in Asian populations. Female predominance (4:1 F:M ratio) Mostly diagnosed in children.	Communication with the biliary tree may be mistaken for fistula to the biliary tree.	Originates in the biliary tree rather than the pancreas.

## Data Availability

The original contributions presented in this study are included in the article. Further inquiries can be directed to the corresponding author.
